# PSORI-CM02 Formula Increases CD4+ Foxp3+ Regulatory T Cell Frequency and Ameliorates Imiquimod-Induced Psoriasis in Mice

**DOI:** 10.3389/fimmu.2017.01767

**Published:** 2018-01-08

**Authors:** Haiming Chen, Huazhen Liu, Chuanjian Lu, Maojie Wang, Xiong Li, Hui Zhao, Yuhong Yan, Wanling Yu, Ling Han, Zhenhua Dai

**Affiliations:** ^1^The Second Affiliated Hospital, Guangzhou University of Chinese Medicine, Guangzhou, China; ^2^Postdoctoral Programme, Guangzhou University of Chinese Medicine, Guangzhou, China; ^3^Guangdong Provincial Hospital of Chinese Medicine, Guangzhou, China; ^4^Guangdong Provincial Academy of Chinese Medical Sciences, Guangzhou, China; ^5^Guangdong Provincial Key Laboratory of Clinical Research on Traditional Chinese Medicine Syndrome, Guangzhou, China; ^6^Key Laboratory for Regenerative Medicine, Ministry of Education, Faculty of Medicine, School of Biomedical Sciences, The Chinese University of Hong Kong, Hong Kong, China; ^7^Kunming Institute of Zoology Chinese Academy of Sciences-The Chinese University of Hong Kong Joint Laboratory of Bioresources and Molecular Research of Common Diseases, Hong Kong, China

**Keywords:** psoriasis, inflammation, immunoregulation, regulatory T cell, PSORI-CM02

## Abstract

Psoriasis is an autoimmune and inflammatory disease, which is estimated to affect 2–3% of the population in the world. PSORI-CM02 is an empirical formula of Chinese medicine optimized from Yin Xie Ling, which is widely used to treat psoriasis in China for decades. However, its antipsoriatic mechanisms are still not well understood. Here, we explored the therapeutic effects of PSORI-CM02 on psoriasis and its mechanisms of action in imiquimod-induced psoriasis-like mouse models and human HaCaT cells. In experiments *in vitro*, PSORI-CM02 significantly inhibited HaCaT cell proliferation in dose-dependent and time-dependent manners. Furthermore, it hindered the progression of HaCaT cell cycle and arrested HaCaT cells at G1 phase. On the other hand, our *in vivo* studies demonstrated that PSORI-CM02 dramatically reduced psoriasis area and severity index scores and lesion temperature in imiquimod-induced psoriatic mice. The antioxidative activities of glutathione, catalase, and superoxide dismutase were increased while oxidative activity of malonaldehyde was markedly decreased after treatments with PSORI-CM02. PSORI-CM02 also suppressed the mRNA expression of proinflammatory cytokines, including TNF-α, IL-6, and IL-17, and lowered their protein levels in the serum as well. In addition, PSORI-CM02 could reduce the expression of IKKα and NF-κB in psoriatic skin tissue. It also upregulated the proportion of CD4+ Foxp3+ regulatory T cells (Tregs) in both lymph nodes and spleens and promoted CD4+ CD25+ Treg proliferation *in vitro*. Taken together, our research demonstrated that PSORI-CM02 inhibited HaCaT cell proliferation by arresting them at G1 phase and alleviated systemic inflammation and psoriasis in mice *via* altering the oxidative/anti-oxidative status, tipping the balance between Th17 responsiveness and CD4+ Foxp3+ Treg generation, and suppressing the expression of proinflammatory cytokines as well as NF-κB signaling.

## Introduction

Psoriasis is an autoimmune and inflammatory dermatologic disease, which affects about 2–3% of population in the world. Psoriatic pathology mainly includes thickening of epidermis, hyperproliferation of keratinocytes and parakeratosis in the epidermis with massive neutrophil infiltration in the dermis (1). Currently, the mechanisms underlying psoriasis have not been fully explored although it has been established that many factors, including genetic factors, environmental factors, immunological mechanisms, new blood vessel formation, lipid metabolism disorders, and unhealthy mentality, bear significant impacts on the occurrence of psoriasis (2). Nowadays, accumulating evidence suggests that patients with moderate or severe psoriasis may increase the risk of other diseases, including obesity, cancer, diabetes mellitus and the metabolic syndrome (3). Meanwhile, current treatments for psoriasis, such as topical therapies, phototherapies and conventional systemic therapies, are not completely satisfied by sufferers principally because of side effects and economic burden (4, 5).

During recent decades, Chinese herbal medicine, one of the traditional Chinese medicine that serves as a complementary and alternative medicine, has been used as popular strategies for treating psoriatic patients and aroused a remarkable and growing interest (6). Our previous researches have shown that Chinese herbal medicine can provide an effective therapy for psoriasis (7–10). PSORI-CM02, which was optimized based on Chinese herbal formula Yin-Xie Ling discovered by well-known professor Guo-Wei Xuan, is a novel formula of Chinese medicine that has been used to effectively treat psoriasis during recent years. PSORI-CM02 consists of five Chinese herbs (Table 1), including *Rhizoma curcumae, Radix paeoniae rubra, Sarcandra glabra, Rhizoma smilacis glabrae*, and *Fructus mume*. Currently, PSORI-CM02 formula is further undergoing a randomized, double-blinded and placebo-controlled clinical trial for treating stable psoriasis vulgaris with a syndrome pattern of blood stasis in our hospital.

**Table 1:** Constituents of PSORI-CM02.

Linnean classification	Botanical origin	Ratio
*Curcumae Rhizoma*	*Curcuma kwangsuensis* S.G. Lee et C.F. Liang	2
*Radix Paeoniae Rubra*	*Paeonia lactiflora* Pall.	3
*Rhizoma Smilacis Glabrae*	*Smilax glabra* Roxb.	5
*Mume Fructus*	*Prunus mume*. (Sieb.)Sieb.etZucc.	2
*Sarcand Raeherba*	*Sarcandra glabra* (Thunb.)Nakai	5

The objective of our current work was to reveal the antiproliferative properties of PSORI-CM02 in human HaCaT cells *in vitro* and the therapeutic effects of PSORI-CM02 on imiquimod-induced murine psoriasis as well as its mechanisms of action. We found that PSORI-CM02 suppressed HaCaT cell proliferation by hindering their cell cycle progression at G1 phase, inhibited the expression of proinflammatory cytokines and NF-κB signaling, upregulated CD4+ Foxp3+ regulatory T cells (Tregs) *in vivo* and promoted their *in vitro* expansion as well while reducing IL-17 production and ameliorating murine psoriasis.

## Materials and Methods

### Animals

BALB/c mice (male, weighing 20 ± 2 g) were purchased from the Center of Laboratory Animals of Southern Medical University (Guangzhou, China). Mice were housed in a standard housing room with controlled temperature (22 ± 2°C), relative humidity (45–55%), artificial light (12 h light/dark cycle), and provided free access to food and water under a specific pathogen-free environment. The animal protocols were approved by the Animal Experimental Ethics Committee of Guangdong Provincial Hospital of Chinese Medicine.

### Chemicals

Minimal essential medium (MEM), fetal bovine serum (FBS), and antibiotics (penicillin–streptomycin) were purchased from Gibco (Carlsbad, CA, USA). Dexamethasone acetate (DXM) was obtained from Shanghai Xinyi Pharmaceutical Factory (Shanghai, China). Imiquimod cream was obtained from Sichuan Mingxin Pharmaceutical Co., Ltd. (Sichuan, China). Eighteen chemical standards, including citric acid, gallic acid, 5-hydroxymethylfurfural, protocatechuic acid, Quercitrin, were obtained from Shanghai Aladdin Biological Technology Co., Ltd. (Shanghai, China) or Sigma-Aldrich (St. Louis, MO, USA).

### Preparation of PSORI-CM02

Five Chinese herbal components (Table 1) contained in PSORI-CM02 formula were purchased from Guangdong Kangmei Pharmaceutical Company Ltd. (Guangdong, China). These herbs were extracted using distilled water and the extract was concentrated and stored for the future study.

### Ultrahigh-Performance Liquid Chromatography (UHPLC) Analysis

Different batches of PSORI-CM02 formula were monitored for quality control purposes by UHPLC method. Briefly, PSORI-CM02 and 18 standards (Table 2) were dissolved with methanol–0.1% formic acid. Chromatographic separation was carried out with an Accela™ UHPLC system, which was comprised of a UHPLC pump and a PDA detector with a scanning from 200 to 400 nm and recorded at 214 nm. The HPLC conditions were set as following: Column: Kintex^®^ C18, 150 mm × 2.1 mm, 2.6 µm particle size (Phenomenax, USA); Mobile phase components: A was water with 0.1% formic acid and B was methanol; Flow rate: 250 µL/min; injection volume: 10 µL; gradient: 0–45 min, linear gradient of 10–35% A, 45–50 min, 35–46% A, 50–60 min, 46–85% A.

**Table 2:** Eighteen chemical constituents identified in PSORI-CM02.

Peak number	Formula	Identification
1	C_6_H_8_O_7_	Citric acid
2	C_7_H_6_O_5_	Gallic acid
3	C_6_H_6_O_3_	5-Hydroxymethylfurfural
4	C_7_H_6_O_4_	Protocatechuic acid
5	C_16_H_18_O_9_	Neochlorogenic acid
6	C_23_H_28_O_12_	Oxypaeoniflorin
7	C_20_H_27_NO_11_	Amygdalin
8	C_16_H_18_O_9_	Chlorogenic acid
9	C_9_H_8_O_3_	p-Coumaric acid
10	C_16_H_16_O_8_	5-O-caffeoylshikimic acid
11	C_11_H_10_O_5_	Isofraxidin
12	C_21_H_22_O_11_	Neoastilbin
13	C_21_H_22_O_11_	Astilbin
14	C_21_H_22_O_11_	Neoisoastilbin
15	C_21_H_22_O_11_	Isoastilbin
16	C_21_H_22_O_10_	Engeletin
17	C_18_H_16_O_8_	Rosmarinic acid
18	C_21_H_20_O_11_	Quercitrin

### Cell Culture

HaCaT cell line was purchased from American Type Culture Collection (Manassas, VA, USA). HaCaT cells were cultured in MEM (Gibico, USA) medium with 10% FBS (Gibico, USA) and 100 mg/mL and 100 IU/mL penicillin/streptomycin in a humidified atmosphere (5% CO_2_, 37°C).

### HaCaT Cell Proliferation Assays *In Vitro*

*In vitro* HaCaT cell proliferation was measured using MTT assays. Briefly, HaCaT cells in logarithmic growth were collected and transferred into a 96-well microplate. After 24 h, PSORI-CM02 was added to each well to make various concentrations (125, 250, 500, and 1,000 µg/mL, respectively) with six replicate wells *per* concentration. After further incubation for 24, 48, and 72 h, 10 µL of 5 mg/mL MTT was added to each well and incubated at 37°C for an additional 4 h. The supernatant then was removed and 100 µL of DMSO was added into each well. The absorbance (A value) was measured at the wavelength of 490 nm. The cell proliferation was presented as an OD value.

### Cell Cycle Analysis

HaCaT cells were placed into six-well plates at 1.0 × 10^6^ cells/well and treated with various concentrations of PSORI-CM02 (125, 250, and 500 µg/mL) for 72 h. The cells were collected, rinsed twice with ice cold PBS and then fixed in 70% ethanol at 4°C overnight. The cells then were subject to a 30-min incubation with 250 µl of RNase A (100 µg/ml) at 37°C and propidium iodide (50 µg/ml, 500 µl) staining for 1 h. Stained cells finally were analyzed *via* a FACS-Calibur flow cytometer (BD Biosciences, San Jose, CA, USA). Three independent experiments were carried out.

### Administration of Drugs

BALB/c mice were randomly grouped into six groups (*n* = 6 *per* group). The control groups were normal mice that were totally untreated. Vehicle and treatment groups, but not control groups, were given topical administration of imiquimod cream to induce psoriasis. Vehicle groups were administrated with distilled water while treatment groups of the mice were orally given DXM (10 mg/kg) or PSORI-CM02 (3, 6, and 12 g/kg, respectively), which was dissolved in distilled water, for a period of seven days. For *in vitro* experiments, control groups were the untreated cells in the media.

### Imiquimod-Induced Psoriasis-Like Mouse Model

According to our previous studies, mice were topically administrated with a dose of 62.5 mg of 5% imiquimod cream applied to a shaved area (3 cm × 2.5 cm) on their back for seven consecutive days. The psoriasis area and severity index (PASI), which comprised parameters for skin erythema, scaling, and thickness, was employed to assess the condition of the psoriasis-like lesion on day 7. Parameters were scored independently on a scale from 0 to 4 according to the clinical signs, in which “0” represents none; “1” denotes slight; “2” means moderate; “3” depicts marked; and “4” indicates very marked clinically.

### Measurement of Mouse Temperature by Infrared Thermal Image

The temperature of mice in each group was determined by the infrared thermal imager (Fluke, USA) in a quiet-state mode and photographs were analyzed by Smart View 3.2 software. Mice were then euthanized and blood samples were collected in the absence of anticoagulants and subjected to centrifugation (3,000 rpm) for 10 min to obtain serum for biochemical analyses.

### Histological Analysis

The dorsal skin of the mice was harvested, fixed in 4% paraformaldehyde in PBS, and embedded in paraffin. Sections (5 µm) were then made and stained with hematoxylin and eosin (H&E) for histological analysis.

### Measurements of Glutathione (GSH), Superoxide Dismutase (SOD), Catalase (CAT), and Malonaldehyde (MDA)

Skin tissues were homogenized in Tris-buffer (20 mM, pH 7.5) on ice using Ultra Turraks Homogenizer (IKA, Germany) and were subject to centrifugation (12, 000 rpm) at 4°C for 10 min. The supernatant was used to measure CAT and SOD activities and GSH and MDA levels. The protein concentration was measured using the Bradford method with BSA serving as a standard. The CAT and SOD activities and GSH and MDA levels were analyzed using commercial assay kits following the manufacturer’s instructions (Jiancheng Company, Nanjing, China).

### Measurements of TNF-α, IL-6, and IL-17 *via* Enzyme-linked Immunosorbent Assay (ELISA) and RT-PCR

To evaluate the serum levels of TNF-α, IL-6, and IL-17, commercially available ELISA kits (eBioscience, USA) were utilized based on the instructions. The absorbance was read at 450 nm with a microplate spectrophotometer (Multiskan GO, Thermo Fisher Scientific, USA). Total mRNA was extracted from skin tissue with Trizol reagents and mRNA was then transcribed to cDNA. The primer sequences were shown in Table 3. The relative mRNA expression levels of cytokines versus GAPDH were measured using an ABI 7500 Fast Real-Time PCR System (Thermo Fisher Scientific, USA).

**Table 3:** Primer sequences of target genes.

Target gene	Primer sequence (5′ → 3′)
TNF-α (forward)	ACTGATGAGAGGGAGGCCAT
TNF-α (reverse)	CCGTGGGTTGGACAGATGAA
IL-6 (forward)	TTCTTGGGACTGATGCTGGT
IL-6 (reverse)	CCTCCGACTTGTGAAGTGGT
IL-17 (forward)	TCAAAGCTCAGCGTGTCCAA
IL-17 (reverse)	TCTTCATTGCGGTGGAGAGTC
GAPDH (forward)	CAGGTTGTCTCCTGCGACTT
GAPDH (reverse)	TATGGGGGTCTGGGATGGAA

### Western Blotting Analysis

Total protein samples from skin lesion tissues were acquired with RIPA lysis buffer followed by centrifugation (12,000 rpm and 5 min) in 4°C. Protein samples were subject to fractionation by SDS-PAGE and electro-transferred to PVD membranes. The membranes were then blocked with 5% (w/v) skim milk in TBS-T containing 0.1% Tween-20 at room temperature for 2 h and subsequently incubated with primary antibody at 4°C overnight. Then, the membranes were washed three times using TBS-T and blotted with a corresponding secondary antibody conjugated with horseradish peroxidase for 1 h. Finally, the protein bands were detected using the enhanced chemiluminescence detection reagents. The band intensity was quantified using Image J software (NIH Image, Bethesda, MD, USA).

### CD4+ Foxp3+ Treg Quantification

Lymph node and spleen cells from mice were prepared after treatments with PSORI-CM02. Cells were stained for surface markers with anti-CD4-PE/CD25-FITC Abs and then an intracellular marker with anti-Foxp3-APC using intracellular fixation/permeabilization kit (eBioscience, San Diego, CA, USA). CD4+ Foxp3 Tregs were analyzed using a FACSCalibur (BD, Biosciences). Since we found that only ~94% of CD4+ CD25+ T cells were FoxP3-positive (Figure S1 in Supplementary Material) in imiquimod-induced psoriatic mice, we quantified CD4+ FoxP3+ rather than CD4+ CD25+ cell population and defined the former as Tregs.

### CD4+ CD25+ Treg Proliferation *In Vitro*

Splenic cells of C57BL/6J were harvested and CD4+ CD25+ T cells were purified by high-speed cell sorting *via* a FACSAria III (BD Biosciences). The purity of the cells was typically >96%. Then, the purified Tregs were labeled with 2 µM CFSE dye (Invitrogen, Karlsruhe, Germany) at 37°C for 10 min, and cultured in 96-well plates (2 × 10^5^ cells/well) coated with anti-CD3/anti-CD28 Abs in complete RPMI-1640 media. PSORI-CM02 (125, 250, and 500 µg/mL) was also added to the medium. After culturing for 4 days, cell proliferation was analyzed *via* FACS analyses.

### Statistical Analysis

The data were statistically evaluated by a one-way analysis of variance followed by Dunnett’s test and denoted as mean values ± SDs. Statistically significant differences were identified as either *P* < 0.05 or *P* < 0.01. All analyses were carried out through SPSS software (version 17.0, SPSS Inc., Chicago, IL, USA).

## Results

### Chemical Profiles of PSORI-CM02

In order to control the quality of PSORI-CM02, we mainly detected eighteen chemical compositions of PSORI-CM02 by UHPLC analysis: citric acid, gallic acid, 5-hydroxymethylfurfural, protocatechuic acid, and so on (Table 2). UHPLC profiling also showed that PSORI-CM02 did not contain rapamycin and cyclosporine (Figure S2 in Supplementary Material).

### PSORI-CM02 Inhibits the Growth of HaCaT Cells

**Figure 1:**
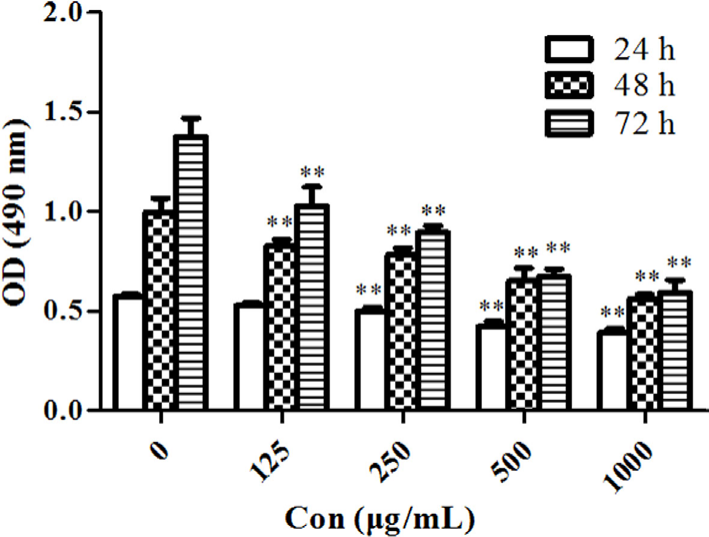
PSORI-CM02 suppresses HaCaT cell proliferation. The proliferation of HaCaT cells *in vitro* was measured *via* MTT assays 24, 48, and 72 h after the cell culture. Data are shown as the mean values ± SDs (*n* = 6, *P < 0.05 and **P < 0.01 vs. the control group: concentration of 0 µg/ml). The results suggested that PSORI-CM02 suppressed HaCaT cell proliferation in a dose-dependent manner.

MTT assay was performed to evaluate the antiproliferative effects of PSORI-CM02 on HaCaT cells *in vitro*. As shown in Figure 1, PSORI-CM02 at various concentrations ranging from 125 to 1,000 µg/mL significantly suppressed HaCaT cell proliferation at all time points (24, 48, and 72 h) in a dose-dependent manner.

### PSORI-CM02 Induces Cell Cycle Arrest in HaCaT Cells

**Figure 2:**
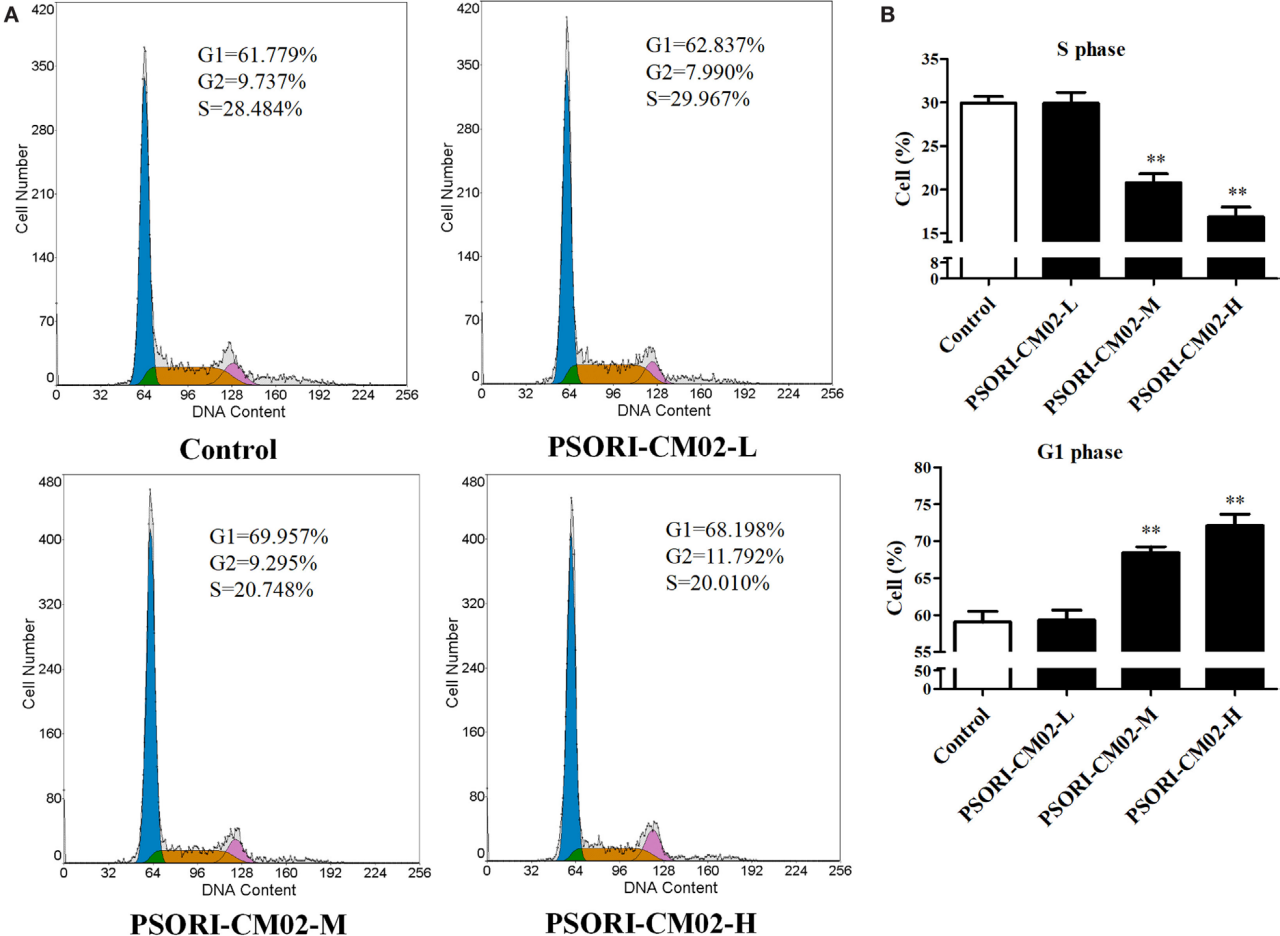
PSORI-CM02 hinders HaCaT cell cycle progression. The cell cycles of HaCaT cells treated with PSORI-CM02 for 72 h were analyzed through flow cytometry using PI staining **(A)**. Histograms exhibited cell cycle distributions (%) in HaCaT cells **(B)**. Results represented the average proportions of S and G1 phases. Data are shown as the mean values ± SDs (*n* = 3, *P < 0.05 and **P < 0.01 vs. control group).

Since cell cycle progression plays an essential role in cell proliferation, flow cytometry was employed to investigate cell cycle distribution in HaCaT cells after treatments of the cells with PSORI-CM02. As shown in Figures 2A,B, there was no significant difference in HaCaT cell cycle progression at all phases between the group treated with low concentration of PSORI-CM02 (L) and control group. Augmenting PSORI-CM02 concentration to 250 (M) or 500 (H) μg/mL increased the frequency of the cells at G1 phase while reducing their percentage at S phase. Taken together, these studies revealed that PSORI-CM02 formula induced HaCaT cell cycle arrest at G1 phase in a dose-dependent manner.

### PSORI-CM02 Alleviates Clinical Symptoms and Reduces Skin Temperature of Imiquimod-Induced Psoriatic Mice

**Figure 3:**
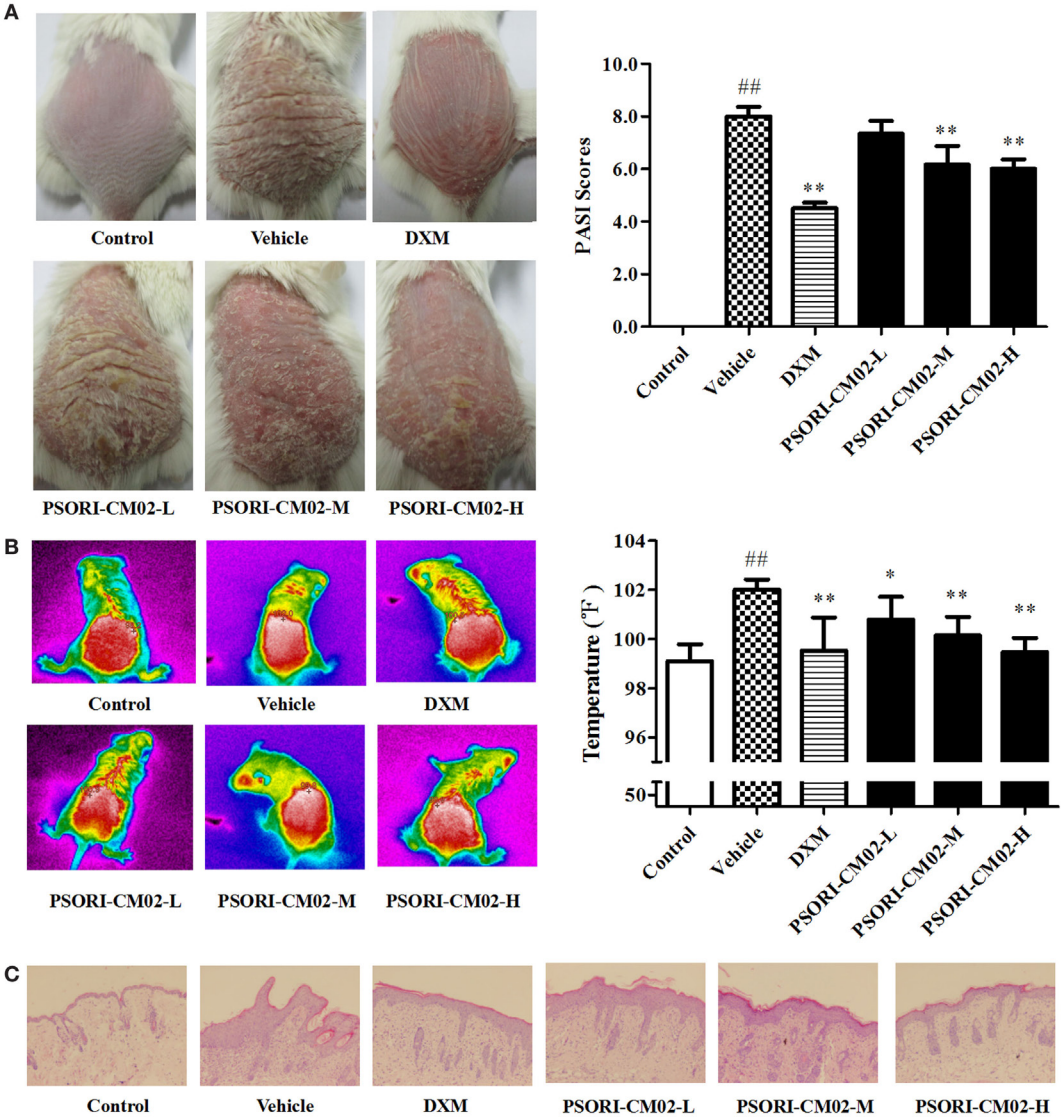
PSORI-CM02 ameliorates murine psoriasis. Shown are macroscopic appearance and the psoriasis area and severity index (PASI) scores of the skin lesions **(A)**, the infrared thermal image of the skin tissue **(B)**, and histological evaluation of the skin tissue *via* H&E staining (magnification 100×) **(C)** in imiquimod-induced psoriasis-like mice treated without or with PSORI-CM02. Data are presented as the mean values ± SDs (*n* = 6, ^#^*P* < 0.05 and ^##^*P* < 0.01 vs. control group, *P < 0.05 and **P < 0.01 vs. vehicle group).

Imiquimod-induced psoriasis-like mouse models were utilized to evaluate the antipsoriatic effects of PSORI-CM02. After treatments with imiquimod cream for three days, the clinical signs of psoriasis-like lesions, including skin erythema, scaling, and thickness, appeared on the shaved dorsal skin, indicating that imiquimod-induced psoriasis-like mouse models were established successfully. Seven days after treatments with PSORI-CM02 or DXM, overall skin lesions were reduced and average PASI scores were decreased significantly compared with the vehicle group, as shown in Figure 3A. Mice treated with high doses of PSORI-CM02 appeared to be healthy except for mild psoriatic skin lesions. Histology demonstrated no liver and kidney injury in these mice (data not shown), suggesting that PSORI-CM02 is not toxic.

Since temperature assessment is one of the important indexes in inflammation, lesion temperature was measured in this study. As shown in Figure 3B, the psoriasis-like lesion temperature was increased significantly (*P* < 0.01 vs. control group) seven days following administration of imiquimod cream while PSORI-CM02 treatments lowered the temperature remarkably compared to the vehicle group.

### Histological Evaluations

Histological examinations *via* H&E staining were performed on the lesion skin seven days after treatments with PSORI-CM02. As depicted in Figure 3C, we found significant pathological changes characterized by increased acanthosis, hyperkeratosis of the epidermis, and abundant inflammatory infiltrates in the skin of imiquimod-induced psoriatic mice. Treatments with either DXM or PSORI-CM02 ameliorated the histological skin lesions, resulting in smoother epidermis, less parakeratosis, and reduced epidermal thickening.

### Effects of PSORI-CM02 on Antioxidative/Oxidative Levels of SOD, GSH, CAT, and MDA

**Figure 4:**
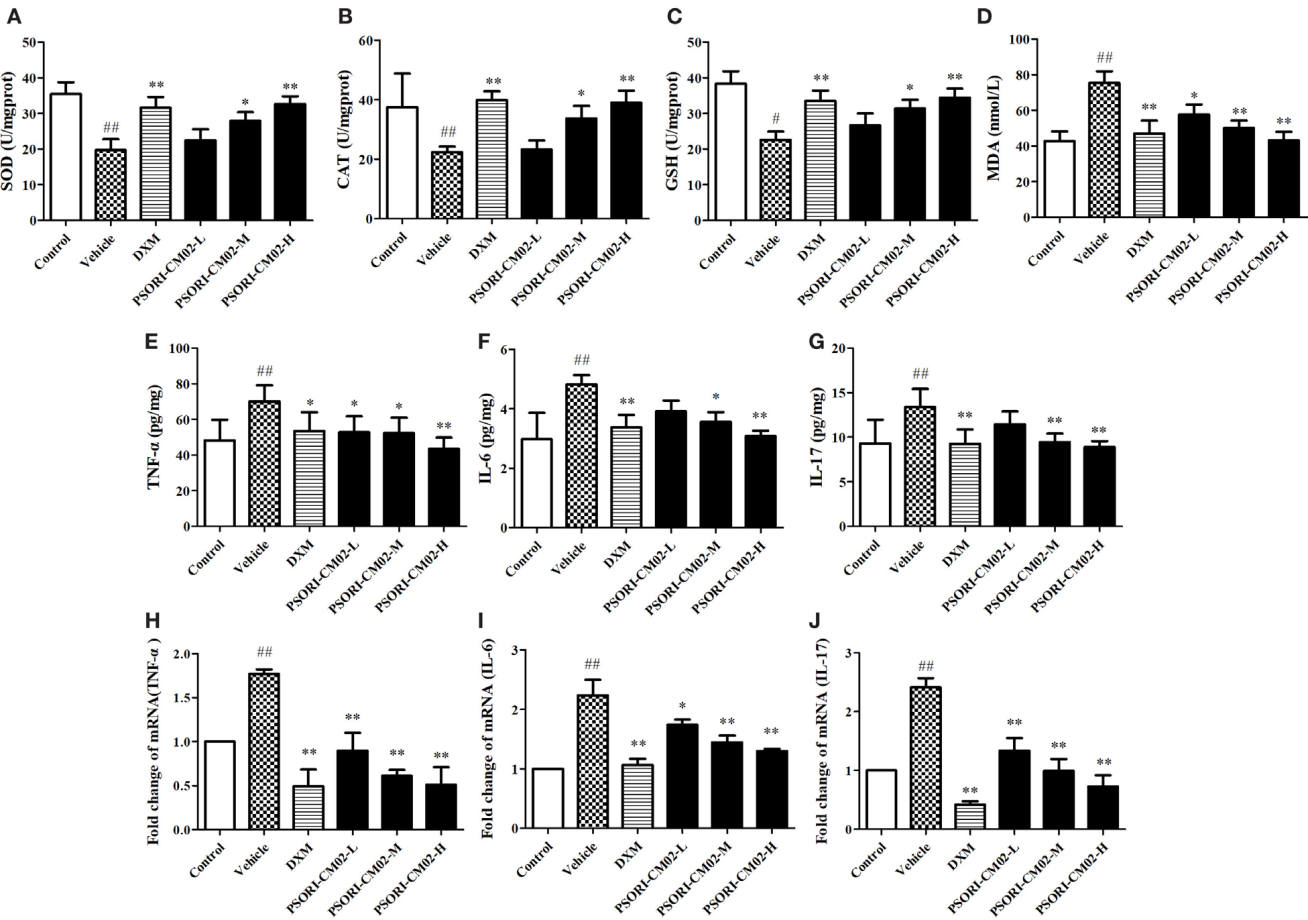
PSORI-CM02 alters oxidative and antioxidative balance and suppresses proinflammatory cytokine expression. Effects of PSORI-CM02 on the activities of superoxide dismutase **(A)**, catalase **(B)**, glutathione **(C)**, and malonaldehyde **(D)** in homogenized skin were determined using enzymatic activity assay kits while the levels of TNF-α **(E)**, interleukin (IL)-6 **(F)**, and IL-17 **(G)** in the serum of imiquimod-induced psoriasis-like mice were evaluated by enzyme-linked immunosorbent assay kits 7 days after PSORI-CM02 treatments. The mRNA levels of TNF-α **(H)**, IL-6 **(I)**, and IL-17 **(J)** in the skin were also determined using RT-PCR. Data shown are the mean values ± SDs (*n* = 6, ^#^*P* < 0.05 and ^##^*P* < 0.01 vs. control group, *P < 0.05 and **P < 0.01 vs. vehicle group).

In this study, SOD, GSH, CAT, and MDA levels in the skin were measured by the corresponding assay kits seven days after treatments. As shown in Figures 4A–D, imiquimod-induced psoriatic mice produced lower levels of CAT, GSH, and SOD, but a higher level of MDA than did control mice. However, upon treatments with PSORI-CM02 at medium or high doses, the antioxidative activities of GSH, CAT, and SOD were elevated significantly (*P* < 0.05 and *P* < 0.01, respectively) compared to the vehicle group. Furthermore, PSORI-CM02 at the doses of 6 and 12 g/kg were shown to obviously reduce an MDA level in the skin tissue (both *P* < 0.01). These results suggested that PSORI-CM02 effectively regulated the oxidative/antioxidative balance toward a more favorable physiological equilibrium in imiquimod-induced psoriatic mice.

### PSORI-CM02 Suppresses mRNA and Protein Expressions of Proinflammatory Cytokines in Imiquimod-Treated Psoriatic Mice

Effects of PSORI-CM02 on proinflammatory cytokine expression were also observed *via* ELISA and RT-PCR seven days after PSORI-CM02 treatment. As shown in Figures 4E–G, the levels of TNF-α, IL-6, and IL-17 in serum in imiquimod-induced psoriatic mice (vehicle) were increased compared to the control group. Although low doses of PSORI-CM02 did not achieve statistical significance, PSORI-CM02 at medium and high doses significantly reduced the levels of proinflammatory cytokines TNF-α, IL-6, and IL-17 in the serum compared to the vehicle group.

The mRNA expressions of TNF-α, IL-6, and IL-17 in skin tissue were also determined using RT-PCR. As shown in Figures 4H–J, the mRNA expressions of TNF-α, IL-6, and IL-17 were augmented after treatments with imiquimod (vehicle) while administration of PSORI-CM02 at all three doses downregulated the mRNA levels of these cytokines compared with imiquimod-treated group without PSORI-CM02 (vehicle).

### PSORI-CM02 Inhibits NF-κB Signaling in the Skin with Psoriasis-Like Lesions

**Figure 5:**
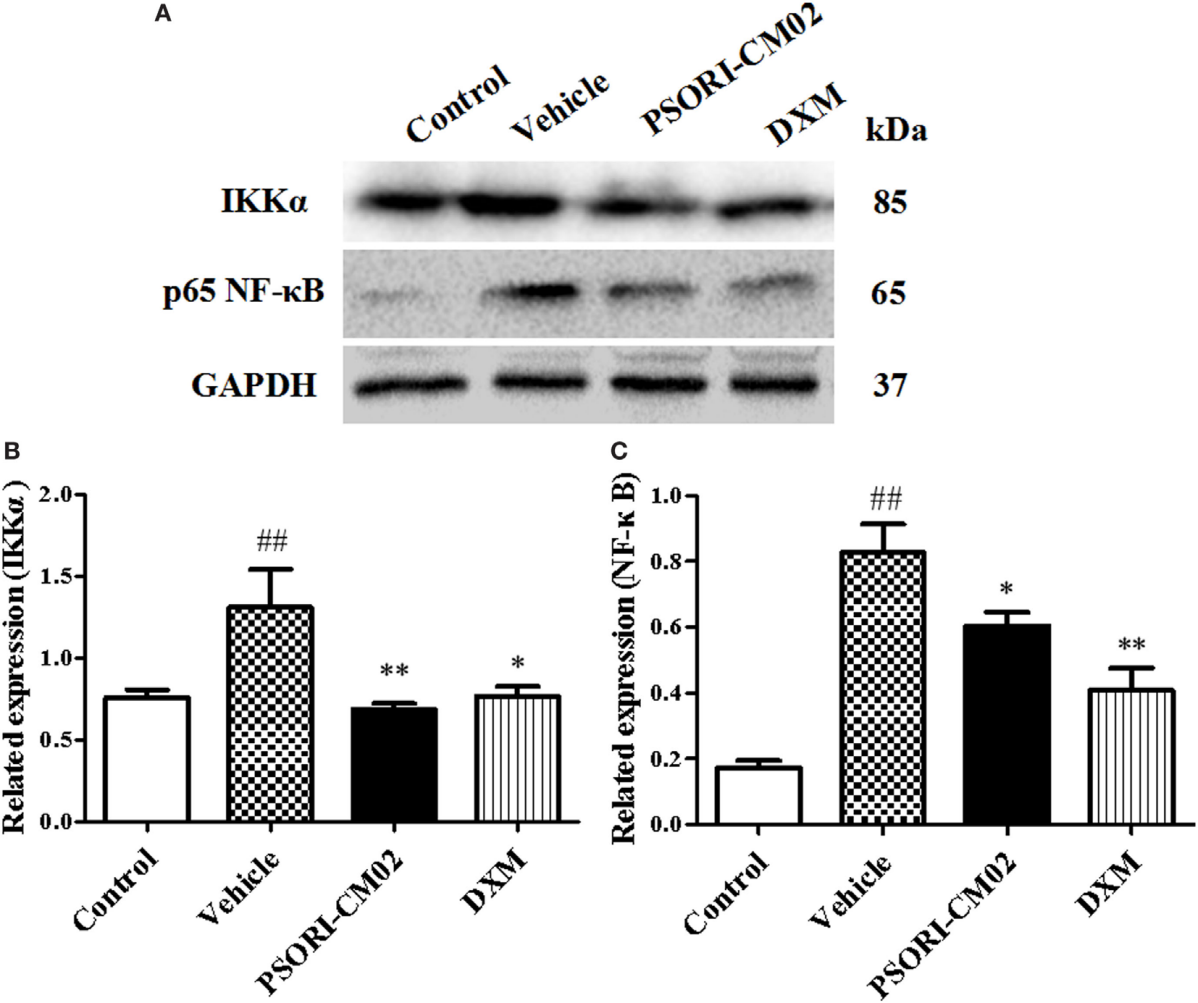
PSORI-CM02 inhibits NF-κB expression in the skin of imiquimod (IMQ)-induced psoriasis-like mice. Impacts of PSORI-CM02 on protein expressions of IKKα and p-65 NF-κB in skin tissue of IMQ-induced psoriasis-like mice were determined using Western blotting analyses seven days after PSORI-CM02 treatments. The expression of IKKα or NF-κB was detected using Western blotting **(A)**. The densitometry analyses of the immunoblotting are shown for IKK **(B)** and NF-κB **(C)**. Data shown are the mean values ± SDs (*n* = 3, ^#^*P* < 0.05 and ^##^*P* < 0.01 vs. control group, *P < 0.05 and **P < 0.01 vs. vehicle group).

Since PSORI-CM02 suppressed proinflammatory cytokine expression, we further explored the mechanisms underlying its antipsoriatic or anti-inflammatory effects. NF-κB signaling is a prominent therapeutic target for treating inflammatory diseases since aberrantly activated NF-κB signaling pathway contributes to inflammatory skin disorders. Thus, western blotting analysis was performed to evaluate the effects of PSORI-CM02 on NF-κB signaling pathway involving protein expression of NF-κB (P65) and IKKα. As shown in Figure 5, the expression of NF-κB and IKKα markedly increased after treatments with imiquimod compared to control group. In contrast, treatments with PSORI-CM02 markedly suppressed the expression of NF-κB and IKKα (*P* < 0.05 and *P* < 0.01, respectively) compared to the vehicle group.

### PSORI-CM02 Upregulates CD4+ Foxp3+ Tregs in Psoriatic Mice

**Figure 6:**
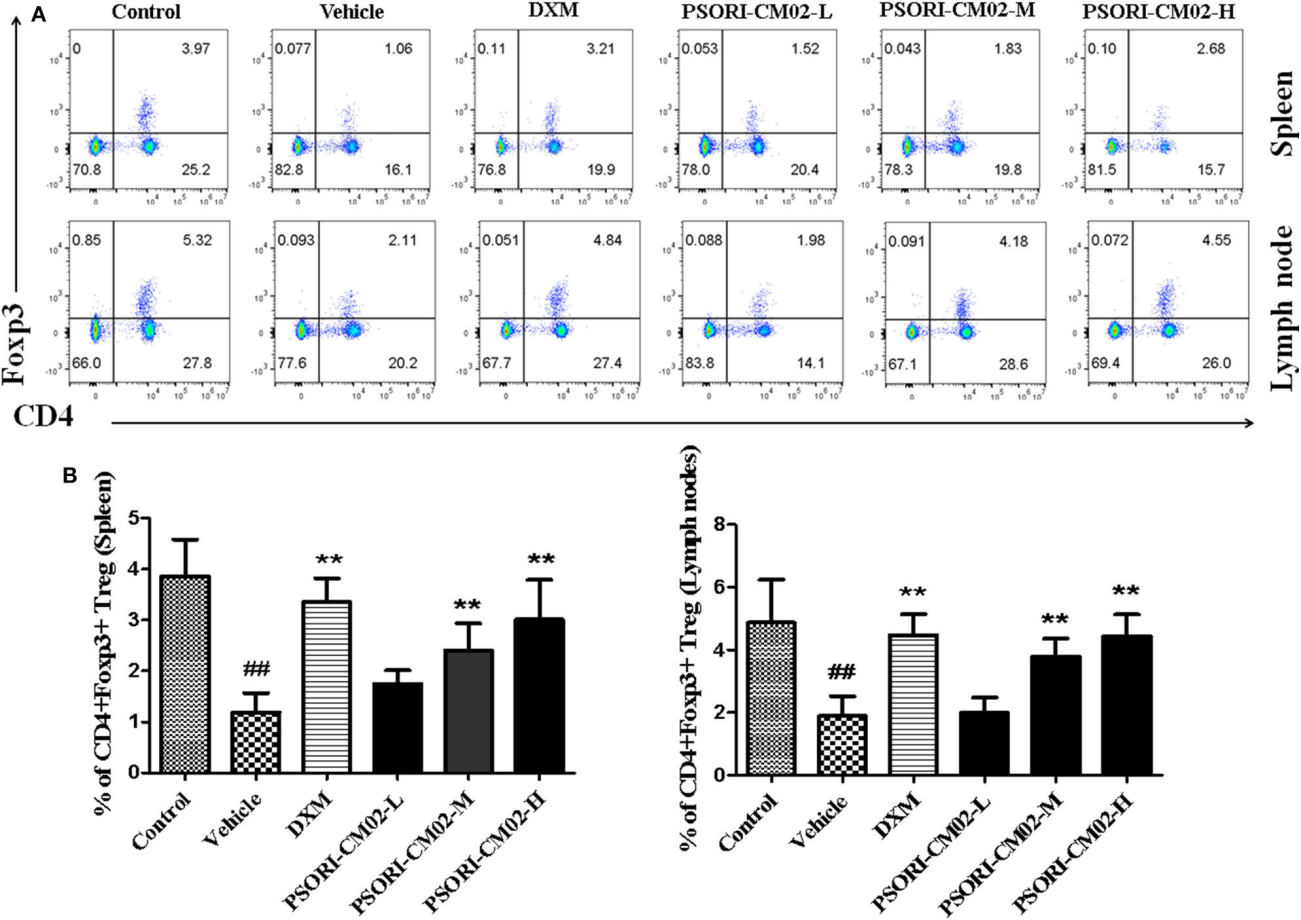
PSORI-CM02 induces CD4+ Foxp3+ regulatory T cells (Tregs) in imiquimod (IMQ)-induced psoriasis-like mice. Effects of PSORI-CM02 on CD4+Foxp3+ Treg frequency in spleens and lymph nodes of IMQ-induced psoriasis-like mice were observed. Spleen and lymph node cells were isolated from IMQ-induced psoriasis-like mice seven days after treatments with PSORI-CM02 or dexamethasone acetate (DXM). To quantify CD4+ Foxp3+ Tregs, cells were stained for CD4 surface and intracellular Foxp3 makers **(A)** and CD4+ Foxp3+ Treg frequency in spleen and lymph node were shown **(B)**. Data shown are the mean values ± SD (*n* = 6, *##P* < 0.01 vs. control group, and **P < 0.01 vs. vehicle group).

Regulatory T cells, a small subset of T cells, play an important role in preventing autoimmune diseases, including psoriasis. Therefore, we determined the frequency of Tregs in lymph nodes and spleens of psoriasis-like mice using FACS analyses seven days after treatments with PSORI-CM02, and the results were displayed in Figure 6. Treatments with either medium or high doses of PSORI-CM02 significantly augmented the frequency of CD4+ Foxp3+ Tregs in both lymph nodes and spleens compared with the vehicle group, although low doses of PSORI-CM02 only slightly increased the proportion of the Tregs.

### PSORI-CM02 Promotes CD4+ CD25+ Treg Cell Proliferation *In Vitro*

**Figure 7:**
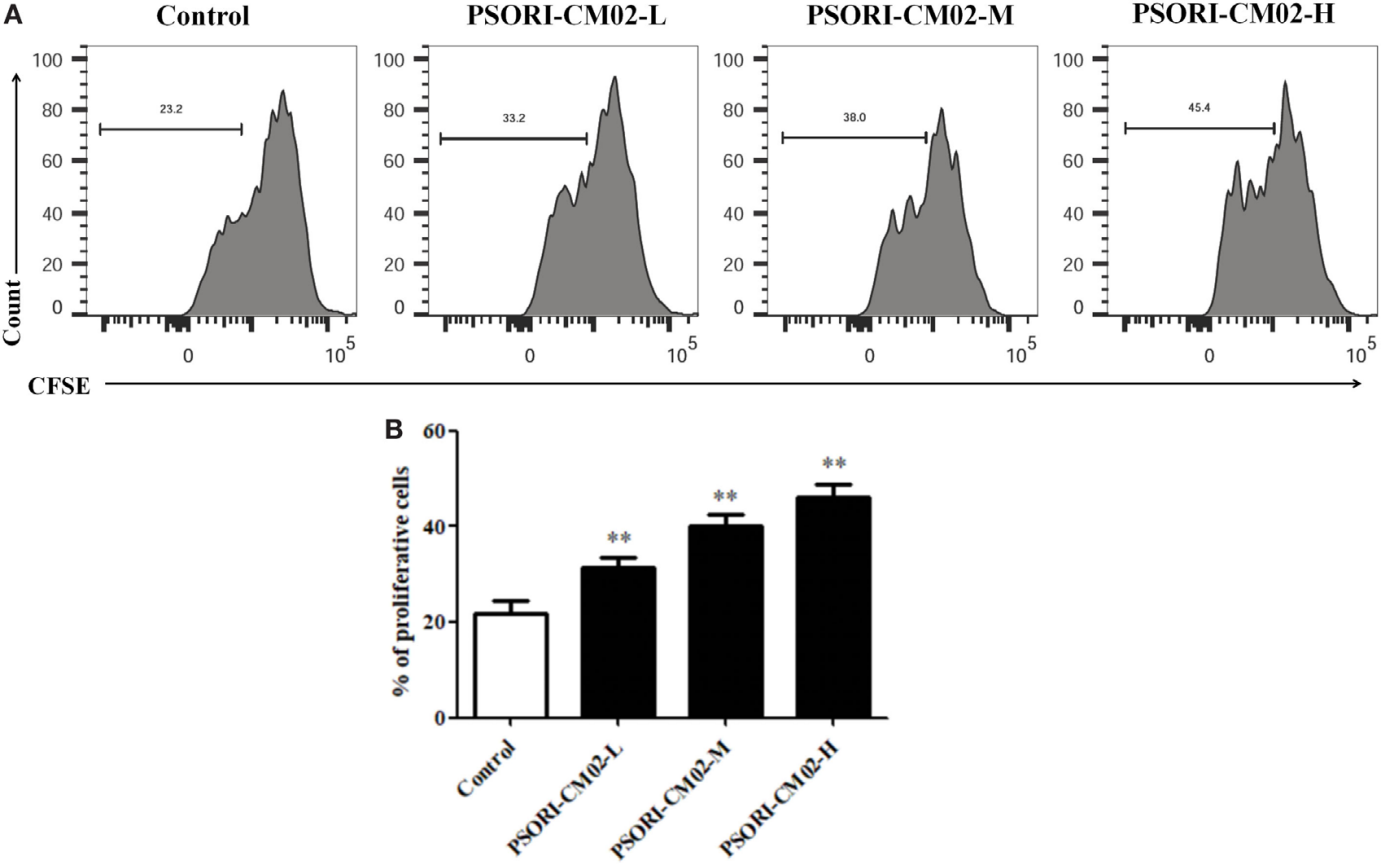
PSORI-CM02 promotes CD4+ CD25+ regulatory T cell (Treg) cell proliferation *in vitro*. Effects of PSORI-CM02 on CD4+ CD25+ Treg cell proliferation *in vitro* were observed. FACS-sorted CD4+ CD25+ Tregs were labeled with CFSE dye and cultured in 96-well plates coated with anti-CD3/anti-CD28 Abs in complete RPMI-1640 media in the absence or presence of PSORI-CM02 (125, 250, and 500 µg/mL). After culturing for 4 days, cell proliferation was analyzed *via* FACS analyses **(A)** and cell proliferation were analyzed **(B)**. Data shown in the bar graph are the mean values ± SDs from three separate experiments (*n* = 3. *P < 0.05 and **P < 0.01 vs. control group).

Given that PSORI-CM02 could upregulate the frequency of CD4+ Foxp3+ Tregs in imuquimod-induced psoriatic mice *in vivo*, we determined its effects on Treg cell proliferation *in vitro*. FACS-sorted CD4+ CD25+ Tregs derived from naïve mice were labeled with CFSE and stimulated with anti-CD3 and anti-CD28 Abs in the absence or presence of PSORI-CM02 for 4 days. As shown in Figure 7, PSORI-CM02 significantly promoted CD4+ CD25+ Treg cell proliferation compared to the control group, suggesting that it can also expand Tregs *in vitro*.

## Discussion

Psoriasis is known as a chronic inflammatory dermatologic disease that affects around 2–3% of the general population (11). Although the causes for psoriasis are not fully understood, its most important pathological features include irregular epidermal hyperplasia, inflammatory infiltration and hyperplastic dermal blood vessels (12). Natural herbal medicine has been verified to be effective in preventing and treating psoriasis by attenuating aberrant proliferation and differentiation of keratinocytes (13, 14) and exerting anti-inflammatory, immunoregulatory (15, 16) and antiangiogenic effects (17). PSORI-CM02 formula is generally used for treating psoriasis in Guangdong Provincial Hospital of Chinese Medicine and has been registered (registration number: ChiCTR-IOR-15006768) for a randomized, double-blinded and placebo-controlled clinical trial. In this study, we endeavored to confirm the therapeutic effects of PSORI-CM02 formula in imiquimod-induced psoriasis-like mice and unravel its possible mechanisms of action *in vivo* as well as its antiproliferative property in HaCaT cells *in vitro*.

It is well known that cell cycle progression mediates cell growth and proliferation and that cell cycle arrest can trigger inhibition of cell proliferation and growth (18, 19). In the current study, the effects of PSORI-CM02 on cell cycle progression was analyzed using flow cytometry, and our results indicated that PSORI-CM02 suppressed HaCaT cell growth by arresting them at the G1 phase (Figure 2B), suggesting that PSORI-CM02 formula may attenuate imiquimod-induced murine psoriasis by inhibiting HaCaT cell growth.

It has been reported that repeatedly topical use of imiquimod in mice results in the influx of various immune cells and hyperplasia of the epidermis, generating a widely used murine model of psoriasis (20). In our study, we established the same model of imiquimod-induced psoriasis and observed that PSORI-CM02 significantly lowered the PASI scores, reduced inflammatory skin temperature, and decreased the epidermal hyperplasia and epidermal thickening compared to the vehicle group, indicating that PSORI-CM02 ameliorates imiquimod-induced murine psoriasis.

We also attempted to reveal the mechanisms underlying the therapeutic effects of PSORI-CM02 on psoriasis. Production of reactive oxygen species (ROS) and weakened antioxidant system take a vital part in the pathogenesis and development of this intractable disease (21). Increased ROS production primarily affects the essential molecules in cells, such as DNA, lipid, protein, and carbohydrate. A balance between antioxidants, including SOD, CAT, and GSH, and oxidants, such as MDA, in normal skin is maintained. Malfunction of the antioxidative system and an increased production of ROS may result in skin diseases, including psoriasis (22–24). In our study, the antioxidative activities of SOD, CAT, and GSH were much lower in imiquimod-treated psoriatic skin than in normal skin whereas the level of oxidative MDA in the psoriatic skin was increased compared to that in the normal skin, which was consistent with the oxidative/antioxidative case of patients with psoriasis (25, 26). Treatments with PSORI-CM02 significantly increased the activities of these antioxidants, including SOD, CAT, and GSH, while decreasing the level of oxidant MDA. Therefore, PSORI-CM02 likely possesses a significant antioxidative feature, resulting in reduced free radical stress and subdued oxidative damage.

Excessive proinflammatory cytokines, such as TNF-α and IL-6, play a key role in the pathogenesis and progress of psoriasis. TNF-α released by multiple types of cells, including activated macrophages, dendritic cells, Th1/Th17 cells, cytotoxic T cells and adipocytes (27, 28), in the serum of patients with psoriasis is essential for the progression of psoriasis (29). It not only activates dendritic cells *via* NF-κB signaling, but also induces expression of adhesion molecules, angiogenic VEGF and other proinflammatory cytokines (30, 31), leading to cell proliferation and inflammation. On the other hand, IL-6 recruits neutrophils (32) and promotes the proliferation of keratinocytes in imiquimod-induced psoriasis-like murine skin (33). In the serum of patients with psoriasis, the level of IL-6 is highly elevated (34). Thus, anti-IL-6 mAb has been proposed to be a novel therapeutic option for the treatment of psoriasis (35).

IL-23/IL-17 axis plays a central role in the development of various autoimmune diseases, including human psoriasis and imiquimod-induced murine psoriasis (20, 36). The number of IL-17-producing cells is elevated in mice topically administered with imiquimod (20, 37, 38). Th17 cells are proposed to be a cardinal source of IL-17 family cytokines, and IL-17 has been deemed as a critical cytokine for the establishment and maintenance of the psoriatic phenotype (39). It promotes inflammation *via* binding the receptor located on keratinocytes, dendritic cells, dermal fibroblasts, and endothelial cells (40). Our results also indicate that PSORI-CM02 ameliorates murine psoriasis by reducing IL-17 production.

NF-κB is a key inflammatory signaling pathway and a critical contributor mediating the pathogenesis and progression of psoriasis (41, 42). NF-κB signaling alters functional states of keratinocytes and immune cells *via* exerting its effects on cellular proliferation, differentiation and apoptosis as well as cytokine and chemokine production (39). When keratinocytes are stimulated with TNF-α, their NF-κB signaling pathway is activated, resulting in the overexpression and release of various proinflammatory cytokines, chemokines and enzymes (43). In our present work, we found that PSORI-CM02 significantly suppressed the expression of NF-κB and IKKα in the skin of imiquimod-simulated psoriatic mice. Thus, PSORI-CM02 exerted its therapeutic effects on murine psoriasis by inhibiting NF-κB signaling pathway.

It has been reported that the imbalance between Tregs and Th17 cells is critical for the pathogenesis and development of psoriasis (44). Tregs are essential for the immune homeostasis and tolerance because of their capability of inhibiting the function of other immune cells and inflammatory responses (45). Therefore, we quantified CD4+ Foxp3+ Tregs in spleens and lymph nodes of psoriasis-like mice *in vivo* and determined the effects of PSORI-CM02 on CD4+ CD25+ Treg proliferation *in vitro*. Our results demonstrated that PSORI-CM02 upregulated the frequency of CD4+ Foxp3+ Tregs *in vivo* and promoted *in vitro* proliferation of CD4+ CD25+ Tregs as well, indicating that these Tregs play a role in attenuation of murine psoriasis by PSORI-CM02. On the other hand, previous studies by Di et al. demonstrated that astilbin ameliorated the inflammation in imiquimod-induced psoriasis-like mice *via* suppressing Th17 cell differentiation and IL-17 secretion (16). In our study, we found that astilbin was one of the main ingredients in PSORI-CM02 formula, and that PSORI-CM02 could inhibit the expression and production of IL-17, which was partially consistent with their studies on astilbin. However, we found that PSORI-CM02 also increased Tregs *in vitro* and *in vivo* and suppressed the expression of NF-κB and IKKα. PSORI-CM02 formula was composed of numerous chemical compositions with 18 major compositions identified already. The therapeutic effect of PSORI-CM02 could be stronger than that of astilbin alone.

In conclusion, we demonstrated that PSORI-CM02 inhibited the keratinocyte proliferation through arresting HaCaT cells at G1 phase. In addition, we found that PSORI-CM02 effectively protected imiquimod-induced psoriasis-like mice from skin lesions by decreasing TNF-α, IL-6, and IL-17 levels, regulating the oxidant/antioxidant status, altering the balance between Th17 response and CD4+ Foxp3+ Treg generation, and inhibiting NF-κB signaling pathway.

## Ethics Statement

This study was carried out in accordance with the recommendations of Chinese national guidelines and institutional review board of Guangdong Provincial Academy of Chinese Medical Sciences. The protocol was approved by the Institutional Animal Care and Use Committee of Guangdong Provincial Academy of Chinese Medical Sciences.

## Author Contributions

Conceived and designed the experiments: CL, ZD, and LH. Performed the experiments: HC, HL, MW, WY, and XL. Analyzed and interpreted the data: HC, YY, and HZ. Revised the data analysis and interpretation: HL and ZD. Wrote the article: HC, HL, and ZD. All authors have read and approved the manuscript.

## Conflict of Interest Statement

The authors declare that the research was conducted in the absence of any commercial or financial relationships that could be construed as a potential conflict of interest.

## Supplementary Material

Figure S1 FoxP3+ regulatory T cell phenotypes. Spleen cells from imiquimod-induced psoriatic mice were stained for CD4, CD25 and FoxP3 and analyzed *via* FACS. Only ~94% of CD4+ CD25+ T cells were FoxP3-positive in imiquimod-induced psoriatic mice.

Figure S2 UHPLC profiling of PSORI-CM02. Eighteen major chemical compositions in PSORI-CM02 formula, including citric acid, gallic acid, 5-hydroxymethylfurfural and protocatechuic acid etc., were detected *via* UHPLC analysis, The results also showed that PSORI-CM02 did not contain conventional immunosuppressive agents cyclosporine and rapamycin.

## Abbreviations

CAT, catalase; DXM, dexamethasone acetate; GSH, glutathione; IMQ, imiquimod; MDA, malonaldehyde; PASI, psoriasis area and severity index; SOD, superoxide dismutase; Treg, regulatory T cell.
