# Corrigendum: PSORI-CM02 Formula Increases CD4+ Foxp3+ Regulatory T Cell Frequency and Ameliorates Imiquimod-Induced Psoriasis in Mice

**DOI:** 10.3389/fimmu.2020.01650

**Published:** 2020-07-29

**Authors:** Haiming Chen, Huazhen Liu, Chuanjian Lu, Maojie Wang, Xiong Li, Hui Zhao, Yuhong Yan, Wanling Yu, Ling Han, Zhenhua Dai

**Affiliations:** ^1^The Second Affiliated Hospital, Guangzhou University of Chinese Medicine, Guangzhou, China; ^2^Postdoctoral Programme, Guangzhou University of Chinese Medicine, Guangzhou, China; ^3^Guangdong Provincial Hospital of Chinese Medicine, Guangzhou, China; ^4^Guangdong Provincial Academy of Chinese Medical Sciences, Guangzhou, China; ^5^Guangdong Provincial Key Laboratory of Clinical Research on Traditional Chinese Medicine Syndrome, Guangzhou, China; ^6^Key Laboratory for Regenerative Medicine, Ministry of Education, Faculty of Medicine, School of Biomedical Sciences, The Chinese University of Hong Kong, Hong Kong, China; ^7^Kunming Institute of Zoology Chinese Academy of Sciences-The Chinese University of Hong Kong Joint Laboratory of Bioresources and Molecular Research of Common Diseases, Hong Kong, China

**Keywords:** psoriasis, inflammation, immunoregulation, regulatory T cell, PSORI-CM02

In the original article, there was a mistake in the legend for Figure 6 as published. Instead of “*n* = 3,” the caption should read “*n* = 6.”

In addition, there was a mistake in Figure 6A as published. The fifth diagram in the lower panel of Figure 6A was mistakenly duplicated from the third diagram in the upper panel. Moreover, we have now used the same quadrants for all groups of Spleen vs. Lymph nodes to increase accuracy. The slight changes in quadrants resulted in slight changes in Figure 6B. The correct Figure 6 and legend appear below.

**Figure 6:**
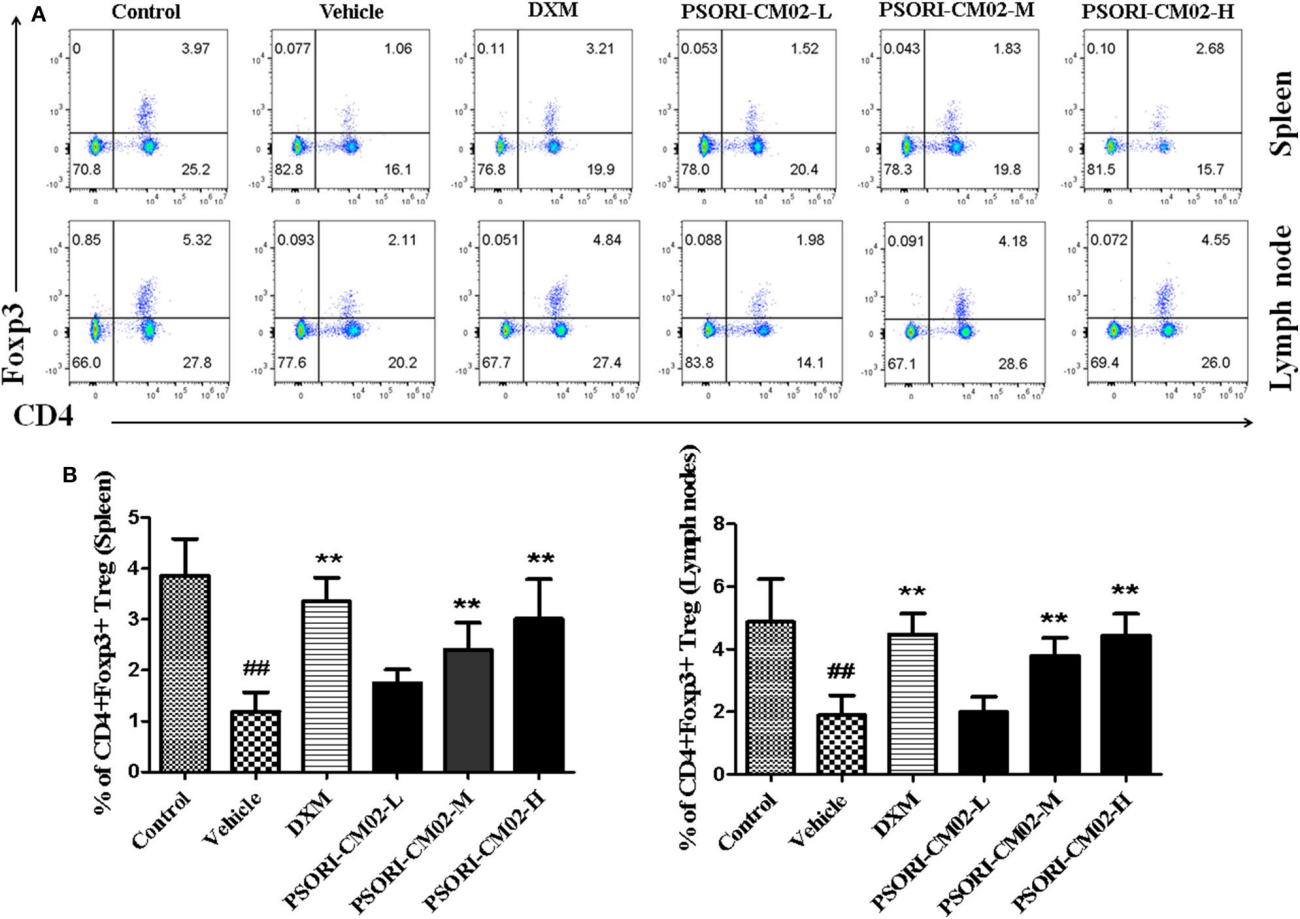
PSORI-CM02 induces CD4+ Foxp3+ regulatory T cells (Tregs) in imiquimod (IMQ)-induced psoriasis-like mice. Effects of PSORI-CM02 on CD4+Foxp3+ Treg frequency in spleens and lymph nodes of IMQ-induced psoriasis-like mice were observed. Spleen and lymph node cells were isolated from IMQ-induced psoriasis-like mice seven days after treatments with PSORI-CM02 or dexamethasone acetate (DXM). To quantify CD4+ Foxp3+ Tregs, cells were stained for CD4 surface and intracellular Foxp3 makers **(A)** and CD4+ Foxp3+ Treg frequency in spleen and lymph node were shown **(B)**. Data shown are the mean values ± SD (*n* = 6, *##P* < 0.01 vs. control group, and ^**^P < 0.01 vs. vehicle group).

The authors apologize for these errors and state that this does not change the scientific conclusions of the article in any way. The original article has been updated.

