# Supplementary figures and images for: PSORI-CM02 Formula Increases CD4+ Foxp3+ Regulatory T Cell Frequency and Ameliorates Imiquimod-Induced Psoriasis in Mice
Source: Front Immunol. 2018 Jan 8;8:1767. doi: 10.3389/fimmu.2017.01767 (PMC5766646; doi:10.3389/fimmu.2017.01767)

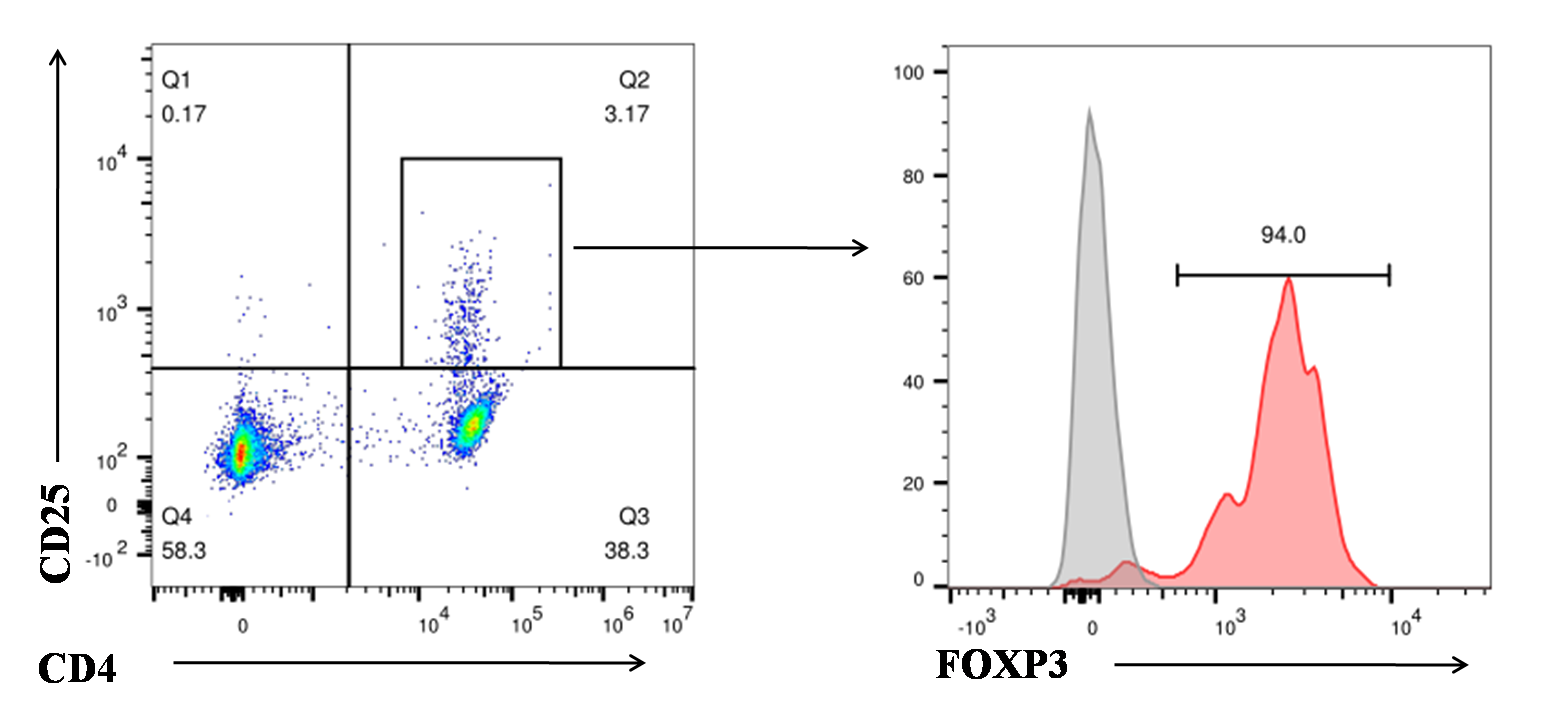

Supplement: Figure S1 — FoxP3+ regulatory T cell phenotypes. Spleen cells from imiquimod-induced psoriatic mice were stained for CD4, CD25 and FoxP3 and analyzed via FACS. Only ~94% of CD4+ CD25+ T cells were FoxP3-positive in imiquimod-induced psoriatic mice. [file Image_1.tif]

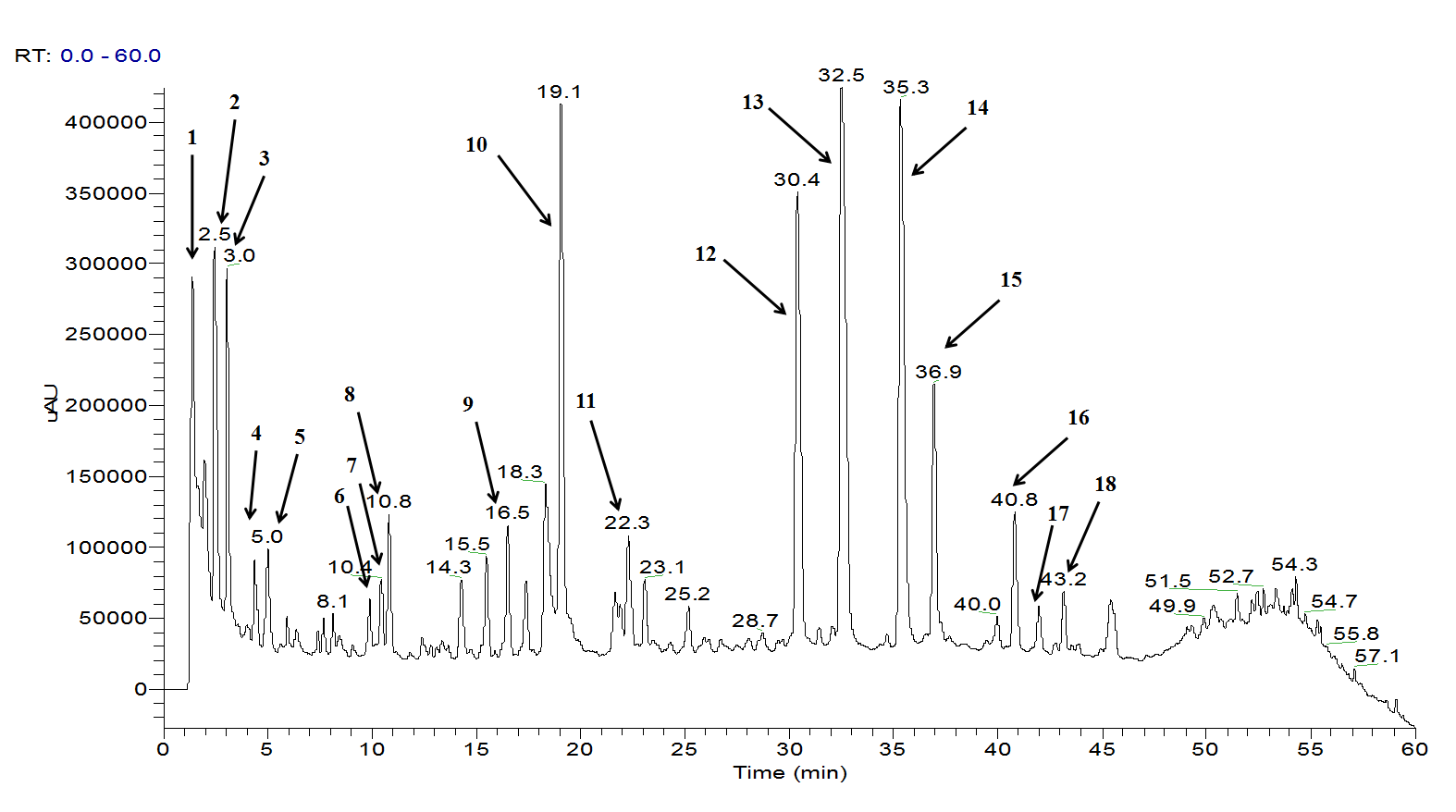

Supplement: Figure S2 — UHPLC profiling of PSORI-CM02. Eighteen major chemical compositions in PSORI-CM02 formula, including citric acid, gallic acid, 5-hydroxymethylfurfural and protocatechuic acid etc., were detected via UHPLC analysis, The results also showed that PSORI-CM02 did not contain conventional immunosuppressive agents cyclosporine and rapamycin. [file Image_2.tif]
